# Laceration of the Cervix Uteri (?) Cured by Homœopathic Treatment

**Published:** 1892-11

**Authors:** A. Quackenbush

**Affiliations:** Buffalo, N. Y.


### LACERATION OF THE CERVIX UTERI (?) CURED BY HOMŒOPATHIC TREATMENT.

EDITOR OF THE HOMŒOPATHIC PHYSICIAN :—I send you a case that I have treated strictly in accord with the law of similia. Mrs. ——, æt. twenty-four, blond, lively, and temperament motive-mental.

Three years ago, during the birth of her first and only child, had a stellate laceration of the cervix uteri.

She was in Brockport, N. Y., and was under the care of some regular (?) physician. He gave her the best scientific (?) treatment known as he told her. Counsel was called. Local treatment applied, but all of no avail.

She wrote to me and asked my advice about her condition, saying that her physicians said an operation would have to be performed, and wanted to know if I would treat her if she would come to her mother's, which was near my old home. I wrote her that I did not consider an operation necessary to bring about a cure, but that to restore the cervix uteri to anything near normal an operation would be necessary, but only after her other trouble was relieved. She delayed coming a few weeks and arrived while I was in Toronto. She wrote me that she was sleepless and that she had been given sleeping medicine. I sent her from Toronto Nux-v.$^{cm}$, and told her that I would be home in two weeks.

CASE.—On vaginal examination to find if there was anything wrong with the cervix I found a stellate laceration, os œdematous and eroded, patient pale and anæmic, emaciation marked, face had a yellowish hue. She could walk across the floor only by supporting herself on chairs or table, and then only with difficulty.

Very despondent about her condition and thought herself incurable. Music makes her weep. Sleepless and wakes about three A. M., and cannot go to sleep again. Gets up in the morning feeling worse than when going to bed. Sleepy in the daytime. Weeps at times. If many were around she was very restless. Terrible pains in the left side of the head and left temple which would almost extort cries ; < from stooping and noises ; > if she could only walk fast, but she is too weak.

Photophobia ; light of lamp hurts her eyes. Smell of food does not agree with her, in fact, it nauseates at times. Her clothes are too tight and has to loosen them often after meals. Wants to put the legs up on a chair as they pain her and feel very uncomfortable when on the floor. Bearing-down pain ; < standing ; < lying or when crossing the legs when standing.

Sexual intercourse could not stand at all, as she had had pain during and a discharge of blood after. Menses were very profuse and amounted to a menorraghia which almost killed her, and would continue for two weeks and stop, but exertion often brought it on again. Flow was dark and clotted. Feet cold and clammy up to the ankles. Chill at different times of the day, but most in the forenoon, with thirst. I could find but little more from her about the chill.

I gave her, July 3d, Sepia$^{1m}$, four doses an hour apart. In a few days felt much improved. Then she received some very depressing news. Wants to be alone and weep. Seems like a ball coming up in the throat, and inclined to be obstinate. Gave Ignat.$^{1m}$, one dose.

July 15th, feels much better in every way, but now has sensation as if drops of cold water were falling on the vertex.

At next menstrual period less loss of blood, and feels better in general. Menses lasted about seven days and less profuse, and now she can walk better and does not bring on the flow.

August 12th.—She went home to-day, taking a good stock of Placebo. Feels better in every way.

September 1st.—Writes me that she has had some return of her menstrual trouble. Studying again carefully, I sent her Sepia$^{50m}$, as it seemed still indicated. Has reported several times and received Placebo.

December 20th.—Sent her Lyc.$^{cm}$, one dose, for the following symptoms: Menorraghia started again. Flow is aggravated every time the bowels move, even if it be a soft stool. Flatulence, has to loosen the clothing, especially after meals, as she feels uncomfortable. Eructations while eating. Flow is clotted like lumps of flesh (Sang.). I did not give Sanguinaria but Lyc., as it seemed to correspond nearest to her condition, and in a week report came that it had entirely ceased, and she had gained strength, and has never had a return of it.

July 4th, 1892.—One year from beginning treatment she came into my office and apologized for not writing to me as she had agreed, and said: "Here I am as sound as ćan be, but you have had only half a chance to cure me, as I only wrote to you when I had to."

She has gained in flesh, and is the picture of health. Says

"she feels perfectly well," but on inquiry I find that she has a little headache at times, like a pressure in the forehead. Aversion to coitus, no enjoyment during, but instead pain, as vagina seems dry. Natr-m.$^{1m}$, four doses.

August 10th.—Reports that these last symptoms are better, and no return of menstrual difficulty from the lacerated cervix.

My reason for presenting this case in detail is that even the homœopathic teachers on this subject say an operation must be performed, and we must not promise to relieve the symptoms under six months.

Here is a case, a clear-cut picture to be sure, but the relief was prompt, and no operation has been performed, although I have advised her to have it to restore the cervix, as I am sure no medicine will overcome a solution of continuity like that. She says: "She is satisfied, as she is, as she fully expected to be under the green grass long ere this."

In this case undoubtedly any tyro could have diagnosed the pathological lesion, and any homœopath worthy of the name would have said that Sepia was the remedy, but as almost every professor of gynæcology in this wide land would have said operate, this woman would have been operated on and a brilliant result, if any had been obtained, would have been attributed to the operation, even though Sepia had been given. Poor old Sepia would have been left out in the cold with no credit for her work. Her work would have been explained away, as a patient did the action of Anthrac. in carbuncle by saying to his physician, "I never saw anything bring anything to a head like that cold water that you told me to apply," when inside of twenty-four hours a blue carbuncle ceased paining and discharged its contents spontaneously. But had Sepia been called in play by the operator he would in all probability used the 3x, as the material mind could not see farther than that.

I am not satisfied with the result, because I have an idea that had I given Sepia higher in the start, say 50M or MM, I would never had to repeat it. In conclusion, I shall say that not all cases are as clear as this, but if we only study our cases more carefully and prove our remedies better we can relieve more of the ills the human flesh is heir to than we can with scissors, scalpel, bistoury, and suture.

There was no local application used at any time for the leucorrhœa, which I forgot to mention was very profuse—thick, yellowish green. A. QUACKENBUSH, M. D.

BUFFALO, N. Y., August 17th, 1892.

